# Investigation of Concrete Shrinkage Reducing Additives

**DOI:** 10.3390/ma15093407

**Published:** 2022-05-09

**Authors:** Martynas Statkauskas, Audrius GRINYS, Danutė Vaičiukynienė

**Affiliations:** Faculty of Civil Engineering and Architecture, Kaunas University of Technology, Studentų Str. 48, LT-51367 Kaunas, Lithuania; audrius.grinys@ktu.lt (A.G.); danute.vaiciukyniene@ktu.lt (D.V.)

**Keywords:** concrete shrinkage, shrinkage reducing additives, quicklime, polypropylene fiber, hemp fiber

## Abstract

This paper analyzes the efficiency of shrinkage reducing additives for the shrinkage deformations of ordinary Portland cement (OPC) concrete and its mechanical properties. OPC concrete was modified with an organic compound-based shrinkage reducing additive (SRA), quicklime, polypropylene fiber, and hemp fiber. It was found that a combination of 2.5% quicklime and 1.5% SRA led to the highest reduction in shrinkage deformations in concrete, and the values of shrinkage reached up to 40.0%. On the contrary, compositions with 1.5% SRA were found to have a significant reduction in compressive strength after 100 freeze-thaw cycles. Hemp fiber did not show a significant shrinkage reduction, but it is an environmentally friendly additive, which can improve OPC concrete flexural strength. Polypropylene fiber can be used in conjunction with shrinkage reducing additives to improve other mechanical properties of concrete. It was observed that 3.0 kg/m^3^ of polypropylene fiber in concrete could increase flexural strength by 11.7%. Moreover, before degradation, concrete with polypropylene fiber shows high fracture energy and decent residual strength of 1.9 MPa when a 3.5 mm crack appears. The tests showed a compressive strength decrease in all compositions with shrinkage reducing additives and its combinations after 28 days of hardening.

## 1. Introduction

Concrete is one of the most common building materials in the world due to its affordable price and particularly good mechanical properties. Nowadays, wide concrete surfaces with wide open surfaces are especially popular in the concrete industry: thin-walled structures, monolithic elements of bridges, or seamless floors. During the hardening of OPC concrete, shrinkage deformations may occur which could lead to various undesirable cracks and fissures. Many scientists are looking for ways to reduce or eliminate the shrinkage of concrete without changing the main properties of concrete [1,2,3,4,5,6,7,8,9,10,11,12,13,14,15].

Shrinkage of concrete can be described as a decrease in volume, regardless of its consistency, which can result from a variety of chemical reactions or a decrease in relative humidity or a slightly saturated porous system. Deformation of materials is often divided into chemical, plastic, carbonization, autogenous, and drying shrinkage and depends on the initial setting time and hydration mechanism [1]. In the early stages of hydration, chemical shrinkage occurs. It is caused by newly formed hydration products with a smaller volume compared to the volume of initial components [2]. Plastic shrinkage is defined as the loss of water by evaporation after the addition of fresh concrete until it becomes hard [3]. Various chemical reactions between carbon dioxide and cement hydration products cause shrinkage of carbonation. In some literature [2,4], autogenic shrinkage of concrete is described as the result of self-drying and chemical processes. This drastically reduces water demand and the water to a cement ratio that is between 0.2 and 0.42. Lack of moisture in the environment causes internal cement dehydration and consequent drying shrinkage.

Many authors report that an effective way to reduce shrinkage deformations is to use fibers and shrinkage-reducing additives that improve the properties of concrete [5,6,7,8,9,10,11,12,13,14,15,16,17,18]. Ullah et al. [5] investigated early age autogenous shrinkage using different types of fibrous materials (steel, plastic, and glass). The study found that a dose of 0.38% fiber volume reduced autogenous shrinkage. Using this number of fibers, it was found that polypropylene fibers had a better effect on autogenous shrinkage compared to steel or glass fiber. Park et al. [6] studied high-performance cement composites containing polypropylene and fiberglass. The findings showed that properties such as compressive strength or tensile strength were higher when polypropylene fiber was used in cement composites compared to glass fiber. The researchers also found that the addition of any of these fibers (>1% by weight) would reduce the shrinkage of the cement composites. It was also found that higher amounts of fiber led to higher efficiency.

Many studies [5,6,7,8,9,10] analyzed the performance of SRA (shrinkage reducing additives) in concrete. Most authors concluded that even a small amount of SRA is sufficient to reduce shrinkage deformations. Zhan and He [7] concluded that SRA is used to delay the hydration reaction of OPC in the early stages due to organic compounds. These compounds reduce the polarity of the OPC and increase the specific surfaces, so more water is needed for hydration. In general, SRA is more effective than geopolymeric materials because the cement matrix does not crack so easily; the cracks are much smaller in width; and large pores of the OPC matrix are reduced. Saliba et al. [8] studied the long-term shrinkage of concrete when SRA was used. The study found that the addition of SRA (1% by weight of OPC) to the concrete reduced the long-term drying shrinkage to 56% and 31% after 7 days of curing at a water to cement ratio of 0.65 and 0.43, respectively. The authors [11] investigated the influence of the propylene glycol effect on concrete shrinkage and mechanical properties using different water/cement ratios. The results showed a decrease in slump and compression with tensile strength (a higher water/cement ratio leads to an even greater decrease). The use of SRA also reduced the free shrinkage (up to 50%) caused by drying.

Many authors [12,13,14,15,16] agree that CaO expansive additives are an excellent way to compensate for concrete shrinkage deformations. CaO additives are a great way to deal with cracks in concrete due to their ability to expand (~90%) by reacting with water. According to Zhao et al. [12], the addition of a CaO additive enriches the hydration process of the cement, especially when a 2% dose is used. The hydration process of the cement intensifies by increasing the temperature of the mixture due to the exothermic reaction of CaO hydration. Polat et al. [13] showed that after 28 days of hardening, the autogenous shrinkage of the concrete was reduced by 42%, 47%, and 80% using an expansive CaO dose of 2.5%, 5.0%, and 7.5% (wt. of cement), respectively. Previous studies [15,16] show that expansive additives such as CaO led to faster hydration, resulting in rapid expansion in the early stages, but much slower in the later stages. Therefore, the use of MgO additives reduces shrinkage at a later stage due to the slow rate of hydration, which results in long-term slow expansion. For this reason, magnesium oxide additives are a better choice for long-term shrinkage compensation. Wang et al. [17] investigated extremely high-quality concrete based on various expansive additives such as highly reactive magnesium oxide, moderately reactive magnesium oxide, and calcium oxide. The study shows that CaO additives better compensate for autogenous shrinkage because they are less sensitive to moisture compared to MgO-based additives.

Çomak et al. [18] studied hemp fibers in reinforced cement mortars with different ratios (0, 1, 2, and 3% by volume of the mix) and lengths (6, 12 and 18 mm). Studies show that 2% of hemp fiber has a significantly higher effect on the mechanical properties of cement mortars. The authors concluded that the best results are obtained using 12 mm long and 2–3% (wt. of mix volume) hemp fiber.

The aim of this study was to investigate the effectiveness of various additives and their combinations in monitoring the influence of shrinkage deformations on concrete mixtures and its mechanical properties. OPC concrete was modified with SRA (organic compound shrinkage reducing additive), quicklime, polypropylene, and hemp fiber to determine the effect of different additives on the properties (density, consistency, air content) of the fresh concrete mix and mechanical properties and durability of hardened concrete (compressive, flexural strength, freeze-thaw resistance, and porosity parameters).

## 2. Materials

Ordinary cement CEM I 42.5 R with the fineness of 390 m^2^/kg was used. The powder of quicklime CL 90 with the fineness of 300 m^2^/kg and reactivity class R5 was incorporated. The amount of quicklime was 1.5% and 2.5% (wt. of OPC) The chemical composition of OPC and quicklime is presented in Table 1.

This experimental study was performed using hemp and polypropylene fibers, and liquid phase shrinkage reducing agents (SikaControl −50) as well. In all mixtures, the same water/cement ratio of 0.53 was applied.

**Figure 1:**
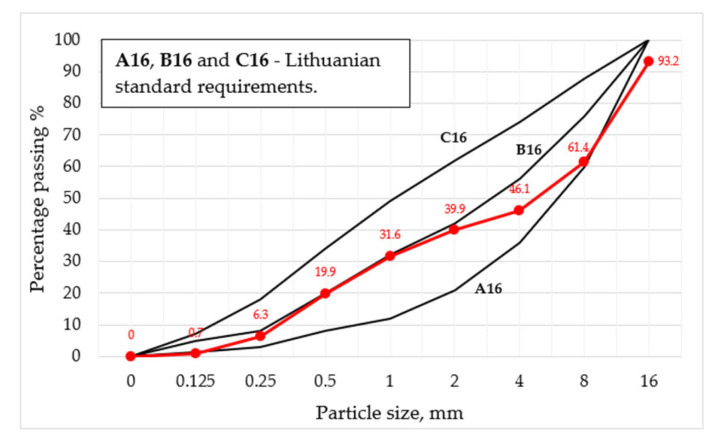
Granulometric curve of concrete aggregates mix.

Coarse aggregate gravel (fr. 4/16 mm) was used with its particle density of 2600 kg/m^3^, while fine aggregate sand (fr. 0/4 mm) with its particle density of 2650 kg/m^3^ was incorporated. In all concrete mixtures, the same amounts of coarse and fine aggregates were added (1006 kg/m^3^ and 870 kg/m^3^, respectively). The particles size distribution of concrete aggregate is shown in Figure 1 (curve in red). The granulometric curve of the mixture for aggregates was formed, and it was determined that the curve did not exceed the Lithuanian Standard LST 1974:2012 [19] requirements.

To reduce the amount of water in concrete, polycarboxylate polymer-based SP (superplasticizer) was used. The admixture has the density of 1.06 ± 0.02 kg/l, pH of 4.4 ± 1, total chloride ion content of <0.1%, and equivalent sodium oxide content of <0.4%. The dosage of superplasticizer was 0.5% (wt. of OPC). SRA based on organic compound has the density of 0.935 ± 0.02 kg/L, total chlorine ion content of <0.1%, and equivalent sodium oxide content of <0.5%. The dosage of SRA was 0.5% and 1.5% (wt. of OPC). Two types of fiber (polypropylene and hemp) were incorporated in the concrete. The density of polypropylene fiber was 0.91 g/cm^3^, melting point of 160–170 °C, tensile strength of 500 MPa, tensile modulus of elasticity of 5.2 GPa, length of 38 mm, and diameter of 0.7 mm. Hemp fiber has a density of 0,16 g/cm^3^, melting point of 140 °C, length of 30–60 mm, and diameter of 0.6 mm. For this research, 3 kg/m^3^ of each fiber were used in concrete.

Under the laboratory conditions, various mixtures (Table 2) were made to examine the efficiency of different additives and their combinations while observing the influence on the shrinkage deformations in concrete mixtures as well as the mechanical properties. 

By combination of initial materials, 11 different mixture compositions with the same water/cement ratio (0.53) were prepared (Table 2).

## 3. Experimental Procedure

The granulometry of aggregates was performed according to EN 933-1 (Figure 1).

Three fresh concrete tests were made: the slump was determined following standard EN 12350-2, air content of compacted fresh concrete according to standard EN 12350-7, and the density of fresh concrete according to standard EN 12350-6.

The concrete mixtures were prepared in the “Zyklos” concrete mixer. The specimens were formed following standard EN 206. The sizes of specimens were standard cubic (100 × 100 × 100 mm) and prism (75 × 75 × 250 mm) which compacted on the vibrational table. The specimens were hydrated for about 20 h in the molds, and, after that they were demolded, cubic specimens were cured in water for 28 days, while prism specimens were cured in air for 90 days.

**Figure 2:**
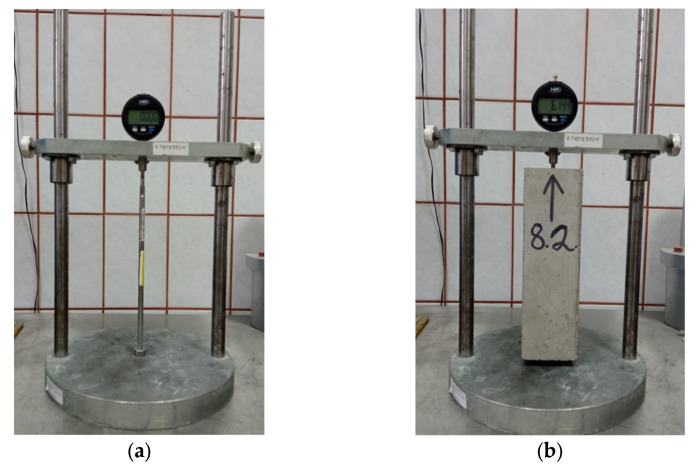
Images of prism length measurement after 90 days; (**a**) setting zero; (**b**) length measurement.

Six hardened concrete tests were made: the density of hardened concrete specimens was determined according to standard EN 12390-6, the compressive strength of hardened concrete according to following standard EN 12390-3, and the flexural strength of hardened concrete according to standard EN 12390-5. Concrete fracture energy was calculated according to CMOD (crack mouth opening displacement) curves. Areas under the CMOD curves were found by using the “Originpro” software [20]. The shrinkage measurement of concrete was determined according to standard EN 12390-16. The concrete prisms length was measured after 3, 7, 14, 28, 56, and 90 days of hardening (Figure 2). Concrete shrinkage strain was calculated following Equation (1):

(1) $$\varepsilon_{cs}\left(t,t_{0}\right)\;=\;\frac{l(t_{0})\;-\;l_{cs}\left(t\right)}{L_{0}};$$

where: $\varepsilon_{cs}\left(t,t_{0}\right)$ is the total shrinkage strain of the specimen at the time t; $L_{0}$ is gauge length; $l(t_{0})$ is the initial length at the time $t_{0}$; $l_{cs}\left(t\right)$ is the length at time t.

The concrete prism weight was evaluated after 3, 7, 14, 28, 56, and 90 days of hardening, and the change in mass of concrete was calculated according to the following Equation (2):

(2) $$X_{cs}\;=\;\frac{W_{cs}\left(t\right)\;-\;W\left(t_{0}\right)}{W\left(t_{0}\right)};$$

where: $X_{cs}$ is total change in mass of the specimen at the time t; $W_{cs}\left(t\right)$ is the initial weight at the time t; $W\left(t_{0}\right)$ is the weight at time t.

**Figure 3:**
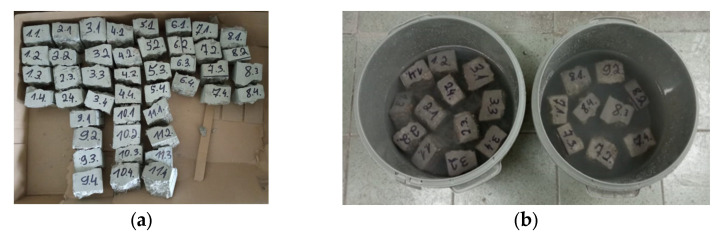

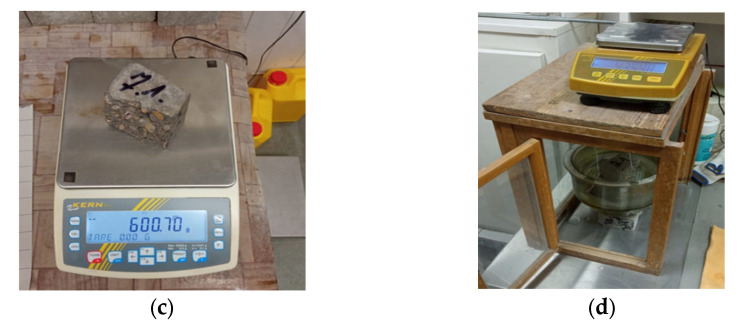
Images of measuring the kinetics for water adsorption; (**a**) dry specimens; (**b**) wet specimens; (**c**) specimen weight measurement; (**d**) specimen weight measurement in water.

Freeze-thaw resistance of concrete was determined by volumetric freezing after immersion in water following Lithuanian standard LST 1428.17:2016 [21]. This test method was used to find out the effect of 100 freeze-thaw cycles on the compressive strength of concrete when different shrinkage reducing additives were incorporated. The freeze-thaw resistance test lasted about 35 days. The porosity parameters were set by measuring the kinetics of water adsorption according to Russian standard GOST 12730.4-78 [22]. This test method was used to find out total, open, and closed porosity of the concrete (Figure 3).

Freeze-thaw resistance and porosity parameters test methods are very well described in the literature [23].

The microstructures of fibers and hardened cement pastes were determined according to scanning electron microscopy with a high-resolution scanning electron microscope (ZEISS EVO MA10).

## 4. Results

### 4.1. Fresh Concrete Test Results

Test results showed an obvious change in the workability of fresh concrete properties (Table 3) when different additives are incorporated.

A reference mixture slump value was 180 mm, resulting in the high workability of the S4 slump class. Most of the batches with earlier mentioned additives have the same or lower slump value than the reference mixture. The slump increased to 190 mm for concrete mixtures with 0.5% and 1.5% (wt. of OPC) of SRA. SRA slightly improved the workability of the concrete mixture because this additive reduces surface tension of the mixing water in the liquid stage, and free water appears in the fresh mixture. A similar explanation is provided in a previous study [5]. The slump slightly decreased in the concrete with 1.5% and 2.5% (wt. of OPC) of quicklime powder; also the workability of the concrete mixture slightly decreased. It was assumed that small amounts of quicklime powder do not significantly affect the slump class, but higher amounts (>2.5% wt. of OPC) of this additive could reduce the slump, because the hydration process of quicklime requires additional water in concrete. Similar observations were made in this study [24]. Concrete with polypropylene fiber decreased the slump value to 110 mm, resulting in the high workability of the S3 slump class. Concrete with hemp fiber decreased slump even more, to 60 mm, resulting in the medium workability of the S2 slump class. The reduction in workability could be explained by the high porosity of the fibers, which could absorb water. Similar observations were made by authors [25,26]. In addition, most of the specimens had acceptable slumps, and there was no detected concrete bleeding or segregation during the experiment.

The fresh concrete density for a reference mixture was 2356 kg/m^3^; air content was 3.4%. Compared to the reference mixture, most of the selected shrinkage reducing additives did not have a significant influence on fresh concrete density. The highest density was obtained in concrete with 1.5 (% wt. of OPC) SRA, and the lowest in concrete with 3.0 kg/m^3^ hemp fiber. The highest air content was also obtained in concrete with 3.0 kg/m^3^ hemp fiber. High air content leads to an assumption that hemp fiber creates interaction between fresh concrete density and air content. The following study [23] explains hemp fiber influence on fresh concrete density and air content. The authors concluded that incorporation of hemp fibers in concrete mixtures notably increases voids’ content under the effect of entrapped air, particularly when the amount of fiber was increased. High air content could be related to the high fiber content, which creates a lot of pores and reduced density and content of pulp in the fresh state as well as poor dispersion of the fibers while the amount of fiber is high. Moreover, a formation of hemp balls is probable, causing heterogeneous parts in the cement matrix and preventing water from entering the concrete, this way making the composite less porous. The densities and air content of all other compositions remained quite similar to the reference mixture.

### 4.2. Hardened Concrete Test Results

#### 4.2.1. Compressive Strength of Concrete

**Figure 4:**
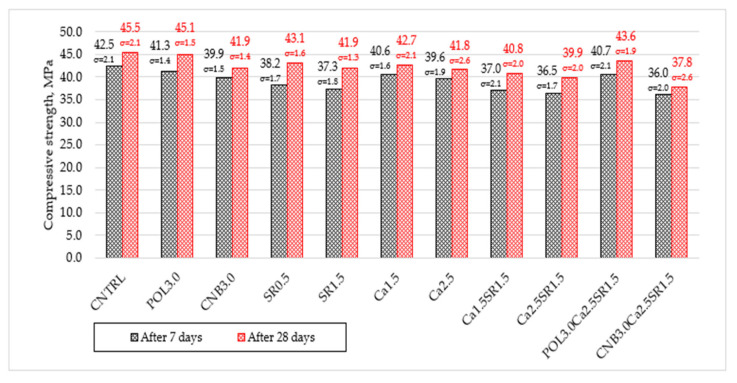
The compressive strength of concrete after 7 and 28 days.

The change in compressive strength of concrete modified with different additives (SRA, quicklime, polypropylene, and hemp fiber) and their combinations is given in Figure 4.

The experiment revealed that the modification of concrete with additives had a significant effect on the compressive strength of the concrete. Reference specimens (CNTRL) without additives had the average compressive strength of 42.5 and 45.5 MPa after 7 and 28 days, respectively. The used additives led to the decrease in compressive strength after 7 and 28 days of hardening. Polypropylene fiber slightly reduces the strength of concrete after 7 and 28 days when compared with the reference mixture. Specimens with 3.0 kg/m^3^ polypropylene fiber (POL3.0) reduced compressive strength by 2.8% and 0.9% after 7 and 28 days, respectively. Similar results were obtained in the study [27] where polypropylene fibers do not particularly affect the compressive strength of concrete.

Specimens with 3.0 kg/m^3^ hemp fiber (CNB3.0) reduced compressive strength by 6.1% and 7.9%. The following study [26] observation confirmed that hemp fibers do not improve concrete compressive strength, which decreases when the addition surpasses 0.25% due to the heterogeneous dispersion of the fibers in the form of balls. The highest decrease in compressive strength, when compared with the reference mixture, was obtained for specimens with the combination of 3.0 kg/m^3^ hemp fiber, 2.5% quicklime, and 1.5% SRA (CNB3.0Ca2.5SR1.5). Compressive strength was reduced by 15.3% and 16.9%.

Specimens with 0.5% shrinkage reducing admixture (SR0.5) reduced compressive strength by 10.1% and 5.3% after 7 and 28 days, respectively. Specimens with 1.5% shrinkage reducing admixture (SR1.5) decreased compressive strength by 12.2% and 7.9%. It can be assumed that the more SRA admixture that is in the concrete, the greater the reduction in compressive strength. the observation was made that SRA slows concrete hydration reactions; in this way, slow formation of calcium silicate hydrate (C-S-H) causes a decrease in compressive strength [11].

The addition of 1.5% quicklime powder (Ca1.5) reduced compressive strength by 4.5% and 6.2% after 7 and 28 days, respectively. Specimens with 2.5% quicklime (Ca2.5) reduced compressive strength by 6.8% and 8.1%. It is assumed that a small amount of quicklime does not cause a huge loss in compressive strength but overdosing (>2.5% wt. of cement) can be expected to reduce the compressive strength quite sharply. The slowdown in the growth in compressive strength may be due to the high expansion stress generated by the hydration of quicklime [11].

The combination of quicklime and SRA reduced compressive strength even more. When 1.5% of quicklime and 1.5% of SRA (Ca1.5SR1.5) were used, compressive strength after 7 and 28 days was reduced by 12.9% and 10.3%, respectively. Moreover, when 2.5% of quicklime and 1.5% of SRA (Ca2.5SR1.5) were used, compressive strength after 7 and 28 days was reduced by 14.1% and 12.3%. It can be assumed that SRA and quicklime acting together can have an even greater negative effect on the compressive strength of concrete than acting alone.

**Figure 5:**
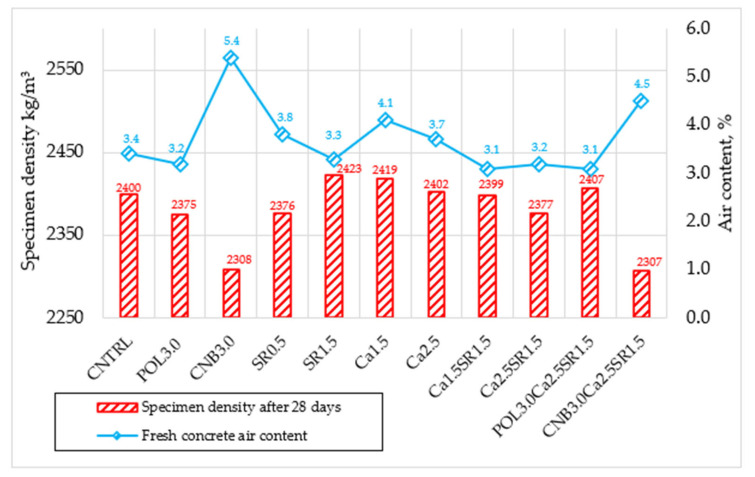
Relationship between specimen density after 28 days and air content.

During the analysis of the densities of the concrete specimens used for the compressive strength test, the average densities of all compositions remained similar. The highest average density of 2423 kg/m^3^ was recorded in the composition SR1.5 and the lowest of 2307 kg/m^3^ in the composition CNB3.0Ca2.5SR1.5. Observing that the densities of the specimens containing 3.0 kg/m^3^ of hemp fiber were lower, it was decided to determine the relationship between the specimen density and fresh concrete air content (Figure 5).

It is assumed that the more air involved in the fresh concrete, the lower the density of the hardened concrete specimen. It was also observed that the specimens with the lowest densities had the lowest compressive strengths. Thus, a correlation between the following indicators occurs: high amount of air content in fresh concrete leads to low density hardened concrete, this way leading to a lower compressive strength. Moreover, there is a possibility that the small differences between the densities could happen technologically due to the potentially unequal compaction times.

#### 4.2.2. Flexural Strength of Concrete

**Figure 6:**
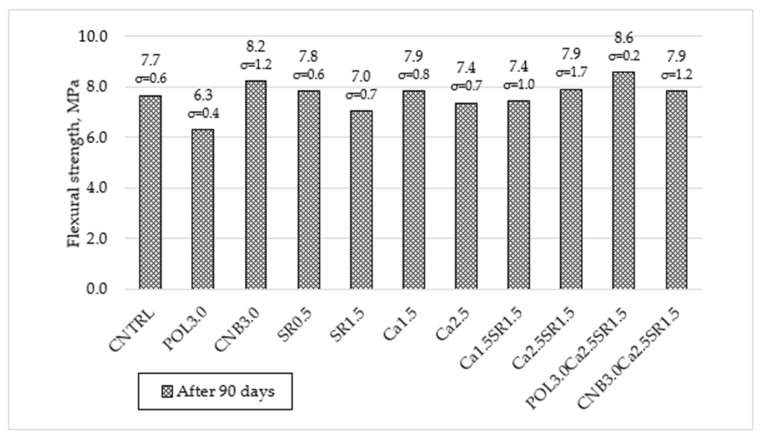
The change in flexural strength of concrete.

The change in flexural strength of concrete modified with different additives (SRA, quicklime, polypropylene, and hemp fiber) and their combinations is given in Figure 6.

**Figure 7:**
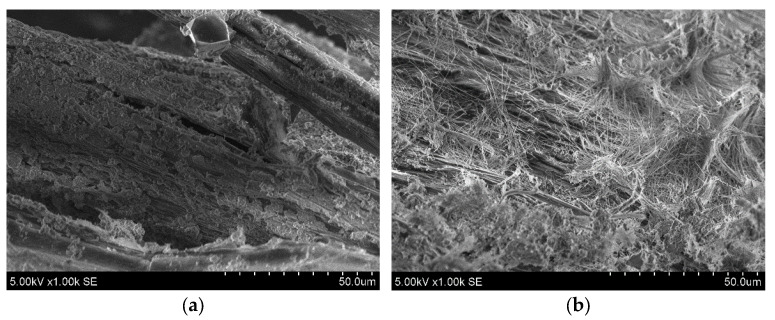
The morphology of polypropylene and hemp fibers; (**a**) surface of polypropylene fiber; (**b**) surface of hemp fiber.

Reference specimens (CNTRL) without additives had the average flexural strength of 7.7 MPa. The incorporation of polypropylene fibers in concrete had contradictory results. Specimens with a combination of 3.0 kg/m^3^ polypropylene fiber, 2.5% quicklime, and 1.5% SRA (POL3.0Ca2.5SR1.5) showed the highest increase in flexural strength of 11.7%. Meanwhile, specimens only with 3.0 kg/m^3^ polypropylene fiber (POL3.0) showed the lowest values of flexural strength results; it decreased by 18.1%. At the design stage of the concrete compositions, an increase in flexural strength was expected to be seen in both compositions with polypropylene fiber, as in the study [25]. A decrease in flexural strength could be related to the poor dispersion of fibers. The interfacial transition zone of polypropylene fibers with OPC paste was investigated by using Scanning Electron Microscope (SEM) analysis (Figure 7a). It was determined that the surface of polypropylene fiber is relatively smooth and homogeneous which could be the reason for the reduction in flexural strength [28].

The use of hemp fibers in concrete had a positive impact on flexural strength results. Specimens with 3.0 kg/m^3^ hemp fiber (CNB3.0) increased flexural strength by 6.5%. The surface of hemp fiber is rougher than the surface of polypropylene fibers (Figure 7b). The reason for the increase in flexural strength could be an increased adhesion between the fiber and OPC-based matrix, due to its rough surface [28].

The SRA shrinkage reducing admixture for 0.5% led to a slight increase in flexural strength by 1.1% when compared with reference specimens, although specimens with 1.5% shrinkage reducing admixture (SR1.5) reduced flexural strength by 9.1%. It is assumed that a small amount of SRA does not negatively affect flexural strength but overdosing (≥1.5% wt. of OPC) can negatively affect concrete flexural strength. Similar flexural strength results were obtained with quicklime powder. Specimens with 1.5% quicklime (Ca1.5) increased flexural strength by 2.6%, and a large amount (2.5%) of quicklime (Ca2.5) decreased flexural strength by 3.9%.

**Figure 8:**
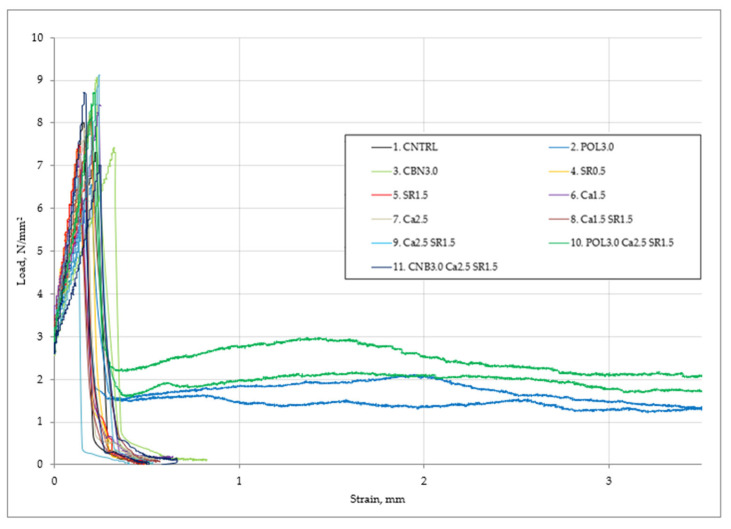
The function of stress and CMOD of concrete.

Concrete potential against fracture was determined by calculating fracture energy, according to study [20]. In this research, fracture energy was calculated to estimate hemp and polypropylene fiber toughness. Fracture energy can be calculated by finding an area under a flexural stress-strain curve until failure. An area under the curve shows the concrete ability to absorb energy. A larger area signifies that concrete can absorb more energy before failure. Area under the CMOD curve was found with software “Originpro” (Figure 8).

Table 4 shows the calculated area according to study [29] for respective specimens. The reference mixture fracture energy is 132–166 N/m. The highest fracture energy was obtained in specimens with 3.0 kg/m^3^ polypropylene fiber: (POL3.0)646and 741 N/m, (POL3.0Ca2.5SR1.5)871 and 1050 N/m. The specimen (POL3.0Ca2.5SR1.5) reached the maximum flexural strength (8.5 MPa), and the specimen cracked but did not experience rapid rupture and continued to withstand the flexural load. The specimen withstood 26.7% of the maximum flexural load at the 0.5 mm crack and 27.2% at the 3.5 mm crack. This phenomenon is explained by the fact that, when the specimen is no longer able to withstand a high flexural load, a crack appears. Under continued loading, the width of the crack gradually increases, but as the crack opens, polypropylene fibers in the concrete structure engage and prevent the specimen from flexing completely. Thus, for this reason, specimens with polypropylene fiber have a residual flexural strength and a high fracture energy. Polypropylene fiber is a great choice to protect concrete from sudden cracking. This fiber is evenly distributed in the concrete, thus creating a kind of 3D grid, so it has excellent reinforcement properties of concrete, which help to take over concrete tensile stresses. Sudden cracking was observed in specimens with 3.0 kg/m^3^ (CNB3.0 and CNB3.0Ca2.5SR1.5) hemp fiber. The specimens did not reinforce concrete properly, and fracture energy was minimal. It is assumed that in order to avoid sudden concrete breakdown, it is advisable to choose polypropylene instead of hemp fiber, because polypropylene fiber absorbs tensile stresses and has a high fracture energy.

#### 4.2.3. Drying Shrinkage of Concrete

**Figure 9:**
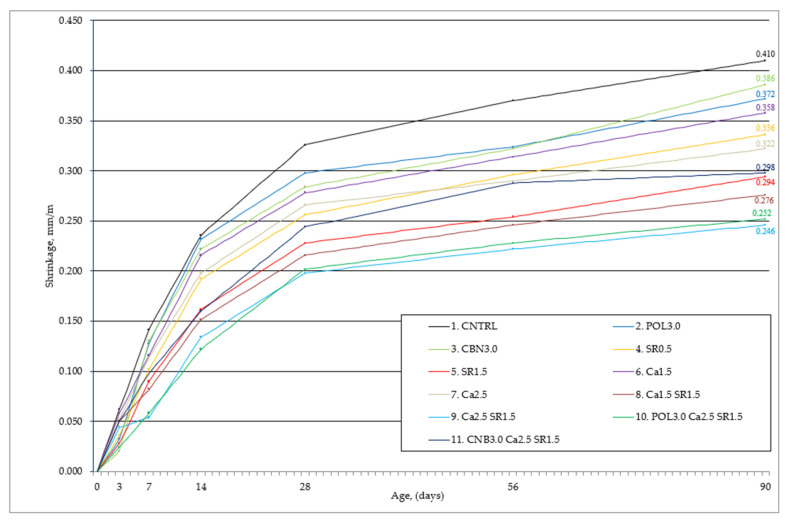
Free-drying shrinkage after 90 days of concrete hardening.

A free-drying shrinkage test was performed on all the specimens, as shown in Figure 9. Length of the specimens was measured at intervals of 1, 3, 7, 14, 28, 56, and 90 days. The tests showed that the reference specimen (CNTRL) average free-drying shrinkage was 0.410 mm/m. It was observed that mostly shrinkage occurs in the early stage of hardening. During the first 28 days, 79.5% of all contractions appeared. Meanwhile, during the rest of the test (29–90 days), the shrinkage of the samples was negligible. The largest concrete shrinkage deformations occur during the first 28 days, as during these days the OPC hydration is most intense. The intensive formation of the hydration products (calcium silicate hydrate) causes a sudden change in the structure of the concrete pores, which becomes the cause of shrinkage. Analyzing the shrinkage results after 90 days of curing, a reduction in shrinkage was observed in all concrete compositions when compared to the reference composition.

The percentages of shrinkage reduction, when compared to reference specimen results, after 7, 28, and 90 days are given in Table 5.

After analyzing the obtained research results, it could be stated that SRA and quicklime had a significant impact on concrete free-drying shrinkage reduction. It is assumed that a small amount of SRA (0.5–1.5% wt. of cement) in the concrete could significantly reduce concrete free-drying shrinkage to 28.3%. Similar experimental results are shown in study [7,8,9,10,11]. Meanwhile, a small amount of quicklime (1.5–2.5% wt. of cement) in the concrete can reduce shrinkage up to 21.5%. Both additives, depending on the amount applied, rapidly reduce the shrinkage of the concrete, and when combined, can show even better results and reduce shrinkage by as much as 40.0%. Theoretically, these two additives could reduce concrete shrinkage even more, but it should be very carefully considered whether too much of these additives would not affect the other mechanical properties of the concrete. Thus, the practical implementation of this idea would require further research, with larger dosages of additives. The use of hemp and polypropylene fiber to reduce concrete shrinkage was not as effective as expected. The use of these fibers alone can reduce shrinkage by only 5.9 and 9.3%. In order to obtain more detailed results, further studies should be performed with different fiber characteristics and dosages.

**Figure 10:**
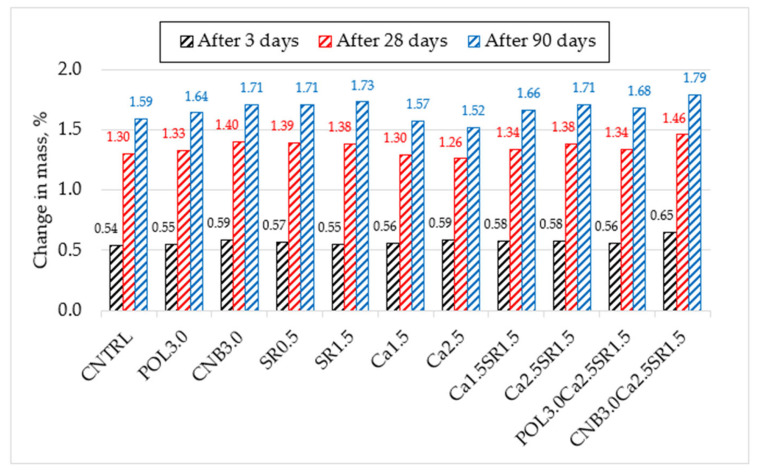
The changes in mass of concrete after 3, 28, and 90 days.

While performing the free-drying shrinkage test, the changes in the mass of concrete specimens were observed, as shown in Figure 10.

Test results showed that after 90 days of hardening, neither concrete composition lost more than 2% of the weight. The change in mass of the reference specimens after 9 days was 1.59%. The highest change in the weight of concrete was observed in the first days; as much as 34.0% of the total weight loss occurred during the first 3 days, and as much as 81.8% after 28 days of hardening. It is assumed that in the first days, the highest amount of free water evaporates from the concrete structure, resulting in the largest changes in the mass of the concrete.

**Figure 11:**
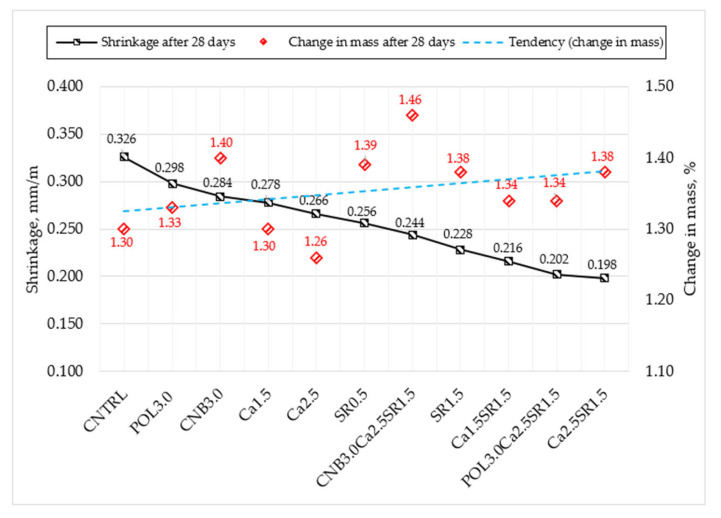
Relationship between shrinkage and change in mass after 28 days.

After noticing that most of the changes in length and weight occur during the first 28 days, it was decided to graph the relationship between these indicators. In the graph (Figure 11), all compositions of concrete mixes are arranged in descending order of shrinkage after 28 days.

The relationship between the shrinkage and change in mass showed that as soon as shrinkage decreases, the change in mass increases. It is assumed that the more shrinkage deformations are reduced, the greater the change in mass can occur.

#### 4.2.4. Freeze-Thaw Resistance of Concrete

**Figure 12:**
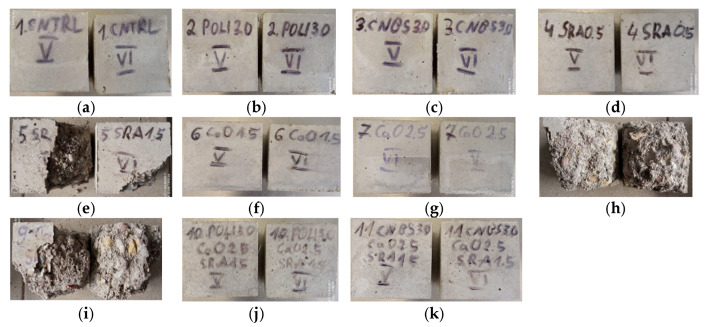
Images of the degradation for concrete specimens after 100 freeze-thaw cycles; (**a**) CNTRL; (**b**) POL3.0; (**c**) CNB3.0; (**d**) SR0.5; (**e**) SR1.5; (**f**) Ca1.5; (**g**) Ca2.5; (**h**) Ca1.5SR1.5; (**i**) Ca2.5SR1.5; (**j**) POL3.0Ca2.5SR1.5; (**k**) CNB3.0Ca2.5SR1.5.

The freeze–thaw resistance of concrete is important for concrete structures which are used in cold countries. The performance of concrete modified with different additives (SRA, quicklime, polypropylene, and hemp fiber) and their combinations after 100 freezing-thawing cycles was investigated. All mixtures’ results were compared with the reference concrete. After 100 cycles, the samples were visually inspected (Figure 12a–k).

**Figure 13:**
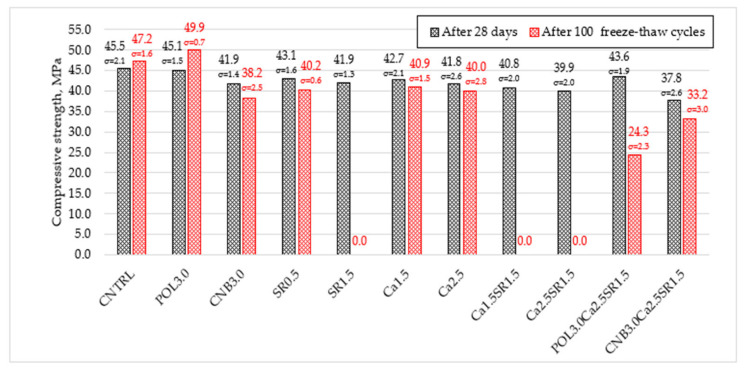
The change in compressive strength of concrete after 100 freeze-thaw cycles.

Examination of freezing-thawing affected specimens showed a tendency for specimens containing 1.5% SRA to crack (Figure 12j,k) or to decompose completely (Figure 12e,h,i). This phenomenon is explained by the fact that a large amount of SRA prevents free water from escaping from the concrete, so when the freeze-thaw cycle occurs, the free water in concrete capillaries freezes and gradually destroys the structure of the specimen, weakening its mechanical properties. The compressive strength of the specimens was tested after 100 freezing and thawing cycles. Figure 13 illustrates compressive strength results after 100 freeze-thaw cycles.

During the analysis of the compressive strength results after 100 freeze-thaw cycles, it was observed that in nine of the eleven compositions, a decrease in the compressive strength occurs compared to the initial compressive strength after 28 days of hardening. The tests showed that the reference specimen (CNTRL) increased by 3.7% (47.2 MPa) from the initial compressive strength (45.5 MPa). POL3.0 specimens increased compressive strength by 10.6%. The hemp fiber (CNB3.0) led to the decrease in compressive strength by 8.8%. Specimens with 1.5% and 2.5% quicklime (Ca1.5 and Ca2.5) decreased compressive strength by 4.2% and 4.3%, respectively. Specimens with 0.5% SRA (SR0.5) compressive strength decreased by 6.7%. Moreover, specimens with hemp fiber, quicklime, and SRA (CNB3.0Ca2.5SR1.5) lost 12.2% of their initial strength. The highest decrease in compressive strength was obtained in specimens with 1.5% SRA, but POL3.0Ca2.5SR1.5). SR1.5, Ca1.5SR1.5, Ca2.5SR1.5 specimens lost 100% of compressive strength due to complete degradation of specimens. Specimens (POL3.0Ca2.5 SR1.5) lost 44.3% of their initial compressive strength. It is assumed that none of aforementioned additives increases concrete resistance to freezing-thawing cycles. High SRA (≥1.5% wt. of cement) content can cause concrete cracking and deterioration of its mechanical properties. In order to use concrete with SRA in places where freezing-thawing cycles occur, it is advisable to incorporate systems that increase freeze-thaw resistance, such as air entrainers or prefabricated air bubbles.

#### 4.2.5. Porosity Parameters of Concrete

By measuring the kinetics of water adsorption, concrete porosity parameters were determined. Total, open, and closed porosity is calculated by using the water adsorption test [30]. The reference specimen adsorbed 4.81% of water after 48 h (Table 6).

CNB3.0 specimens adsorbed the highest amount of water 21.6%, and Ca2.5 specimens adsorbed the lowest amount of water 6.2% by comparing with the reference composition. The relationship between specimen densities and water adsorption was observed. It assumed that concrete with higher densities has the lowest water adsorption. Thus, the lower the density of the concrete specimens, the easier it is for the concrete to adsorb water, so it is necessary to increase the density of the concrete or the closed porosity to reduce the water adsorption.

**Figure 14:**
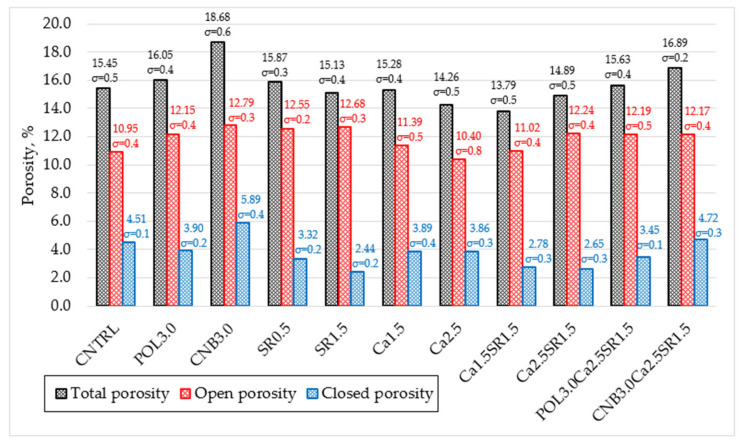
Total, open, and closed porosity of concrete.

The types of concrete for porosity results are illustrated in Figure 14. The total porosity of the reference specimen is 15.45% of which 70.9% is open porosity and 29.1% is closed porosity. CNB3.0 specimens had the highest porosity: total 18.68%, open 12.79%, and closed 5.89%. Compared to the reference specimen porosity, concrete with hemp fiber (CNB3.0) increased total porosity by 20.9%, open by 16.8%, and closed by 30.6%. It can be assumed that hemp fiber is a light, low-density material that tends to adsorb water, resulting in high porosity. The lowest total porosity of 13.79% was obtained in the specimen with a combination of 1.5% quicklime and 1.5% SRA (Ca1.5SR1.5) compared to the reference concrete total porosity that was decreased by 10.7%. The lowest open porosity of 10.4% was obtained in the specimen with 2.5% quicklime (Ca2.5); open porosity was decreased by 5.0%. The lowest closed porosity of 2.44% was obtained in the specimen with 1.5% SRA (SR1.5); closed porosity was decreased by 45.9%. After analyzing the obtained research results, it could be stated that concrete specimens with high open porosity are able to adsorb the highest amount of water, and specimens with higher closed porosity are able to withstand a greater number of freeze-thaw cycles.

## 5. Conclusions

The following conclusions were drawn from the research:

The results of the experiment showed a significant reduction in concrete shrinkage when local quicklime powder is used as a shrinkage reducing additive. It was found that a small amount of local quicklime powder (>2.5% wt. of OPC) can reduce concrete shrinkage up to 21.5%. In addition, interactions with other shrinkage-reducing additives, such as SRAs based on organic compounds, can achieve even better shrinkage-reducing results. The combination of 1.5% SRA and 2.5% quicklime, which reduced shrinkage deformations up to 40%, was recommended. The drying shrinkage is closely related to physical and mechanical properties of concrete as well.

The compressive strength test showed a strength decrease in all investigated compositions after 28 days of hardening. The highest decrease about 16.9% was obtained in composition (CNB3.0Ca2.5SR1.5) and the lowest 0.9% in composition (POL3.0). Flexural strength tests showed the highest strength increase (11.7%) in concrete with 3.0 kg/m^3^ polypropylene fiber. Meanwhile, the lowest strength had (POL3.0Ca2.5SR1.5) specimens with the 2.5% quicklime and 1.5% SRA addition. Moreover, this concrete had the highest fracture energy and residual strength of 1.9 MPa when a 3.5 mm crack appeared.

The parameters of water adsorption kinetics show that concretes with higher densities adsorb less water, while concretes with high closed porosity withstand higher amounts of freeze-thaw cycles. The water adsorption property of hemp fiber has been found to have a significant effect on the properties of concrete. Compared to the reference concrete, the use of 3.0 kg/m^3^ hemp fiber reduced concrete workability, but increased air content, which is the main factor in the increase in closed porosity of hardened concrete. Compositions with 1.5% SRA were found to have a significant reduction in compressive strength after 100 freeze-thaw cycles. This observation is closely related to the morphology of hemp fibers.

## Figures and Tables

**Table 1:** Oxide compositions of OPC and calcium quicklime powder, %.

Oxide	CEM I 42.5 R	CL 90 Q
CaO	63.2	95.91
SiO_2_	20.4	0.52
Al_2_O_3_	4.0	0.06
Fe_2_O_3_	3.6	0.05
MgO	2.4	0.29
K_2_O	0.9	-
Na_2_O	0.2	-
SO_3_	3.1	-
Loss on ignition (%)	2.15	1.04

**Table 2:** The mixtures of initial materials for 1 m^3^ concrete mixtures.

Notation	CEM I 42.5 R, kg	Water, L	Gravel (4/16), kg	Sand (0/4), kg	CL 90 Q, kg	Fiber, kg		Additives, (% wt. of OPC)	
						Polypropylene	Hemp	SP	SRA
Reference	319	169	1006	870	-	-	-	0.5	
POL3.0	319	169	1006	870	-	3.00	-	0.5	
CNB3.0	319	169	1006	870	-	-	3.00	0.5	
SR0.5	319	169	1006	870	-	-	-	0.5	0.5
SR1.5	319	169	1006	870	-	-	-	0.5	1.5
Ca1.5	319	169	1006	870	4.79	-	-	0.5	
Ca2.5	319	169	1006	870	7.98	-	-	0.5	
Ca1.5SR1.5	319	169	1006	870	4.79	-	-	0.5	1.5
Ca2.5SR1.5	319	169	1006	870	7.98	-	-	0.5	1.5
POL3.0Ca2.5SR1.5	319	169	1006	870	7.98	3.00	-	0.5	1.5
CNB3.0Ca2.5SR1.5	319	169	1006	870	7.98	-	3.00	0.5	1.5

**Table 3:** Fresh concrete properties.

Notation	Slump, mm	Density kg/m^3^	Air Content, %
Reference	180	2356	3.4
POL3.0	110	2354	3.2
CNB3.0	60	2303	5.4
SR0.5	190	2349	3.8
SR1.5	190	2368	3.3
Ca1.5	170	2344	4.1
Ca2.5	160	2345	3.7
Ca1.5SR1.5	190	2365	3.1
Ca2.5SR1.5	180	2356	3.2
POL3.0Ca2.5SR1.5	120	2345	3.1
CNB3.0Ca2.5SR1.5	70	2310	4.5

**Table 4:** Fracture energy used to break the specimens.

Notation	Work, J	Fracture Energy, N/m	Residual Flexural Strength at 0.5 mm, MPa	Residual Flexural Strength at 3.5 mm, MPa
CNTRL	1.19	132	0	0
	1.49	166	0	0
POL3.0	6.67	741	1.64	1.36
	5.81	646	1.55	1.32
CNB3.0	1.99	221	0.36	0
	1.83	203	0.07	0
SR0.5	1.15	128	0	0
	1.40	156	0	0
SR1.5	1.18	131	0	0
	1.12	124	0.05	0
Ca1.5	1.20	133	0	0
	1.72	191	0.17	0
Ca2.5	1.22	136	0.15	0
	1.25	139	0.16	0
Ca1.5SR1.5	1.03	114	0.04	0
	1.66	184	0.11	0
Ca2.5SR1.5	0.76	84	0	0
	1.61	179	0.08	0
POL3.0Ca2.5SR1.5	9.45	1050	2.27	2.10
	7.84	871	1.73	1.73
CNB3.0Ca2.5SR1.5	1.26	140	0.04	0
	1.52	169	0.21	0

**Table 5:** The shrinkage reduction of concrete specimens after 7, 28, and 90 days.

Notation	Shrinkage Reduction (%) After:		
	7 Days	28 Days	90 Days
CNB3.0	8.5	12.9	5.9
POL3.0	9.9	8.6	9.3
Ca1.5	18.3	14.7	12.7
SR0.5	28.2	21.5	18.0
Ca2.5	19.7	18.4	21.5
CNB3.0Ca2.5SR1.5	31.0	25.2	27.3
SR1.5	36.6	30.1	28.3
Ca1.5SR1.5	42.3	33.7	32.7
POL3.0Ca2.5SR1.5	59.2	38.0	38.5
Ca2.5SR1.5	62.0	39.3	40.0

**Table 6:** Durability parameters of hardened concrete.

Notation	Water Adsorption, %	Concrete Density, kg/m^3^	K_f_	Predicted Cycles
CNTRL	4.81	2274	4.58	715
POL3.0	5.38	2258	3.57	573
CNB3.0	5.85	2188	5.12	770
SR0.5	5.55	2260	2.94	468
SR1.5	5.56	2283	2.14	327
Ca1.5	5.00	2279	3.79	609
Ca2.5	4.51	2306	4.13	658
Ca1.5SR1.5	4.75	2319	2.80	445
Ca2.5SR1.5	5.35	2289	2.40	376
POL3.0Ca2.5SR1.5	5.37	2270	3.14	503
CNB3.0Ca2.5SR1.5	5.44	2236	4.31	683

## Data Availability

The data presented in this study are available on request from the corresponding author.
